# Extraocular Motor System Exhibits a Higher Expression of Neurotrophins When Compared with Other Brainstem Motor Systems

**DOI:** 10.3389/fnins.2017.00399

**Published:** 2017-07-11

**Authors:** Rosendo G. Hernández, Silvia Silva-Hucha, Sara Morcuende, Rosa R. de la Cruz, Angel M. Pastor, Beatriz Benítez-Temiño

**Affiliations:** Departamento de Fisiología, Facultad de Biología, Universidad de Sevilla Sevilla, Spain

**Keywords:** brain-derived neurotrophic factor, neurotrophin-3, nerve growth factor, extraocular motoneurons, facial, hypoglossal, cranial muscles, amyotrophic lateral sclerosis

## Abstract

Extraocular motoneurons resist degeneration in diseases such as amyotrophic lateral sclerosis. The main objective of the present work was to characterize the presence of neurotrophins in extraocular motoneurons and muscles of the adult rat. We also compared these results with those obtained from other cranial motor systems, such as facial and hypoglossal, which indeed suffer neurodegeneration. Immunocytochemical analysis was used to describe the expression of nerve growth factor, brain-derived neurotrophic factor and neurotrophin-3 in oculomotor, trochlear, abducens, facial, and hypoglossal nuclei of adult rats, and Western blots were used to describe the presence of neurotrophins in extraocular, facial (buccinator), and tongue muscles, which are innervated by the above-mentioned motoneurons. In brainstem samples, brain-derived neurotrophic factor was present both in extraocular and facial motoneuron somata, and to a lesser degree, in hypoglossal motoneurons. Neurotrophin-3 was present in extraocular motor nuclei, while facial and hypoglossal motoneurons were almost devoid of this protein. Finally, nerve growth factor was not present in the soma of any group of motoneurons, although it was present in dendrites of motoneurons located in the neuropil. Neuropil optical density levels were higher in extraocular motoneuron nuclei when compared with facial and hypoglossal nuclei. Neurotrophins could be originated in target muscles, since Western blot analyses revealed the presence of the three molecules in all sampled muscles, to a larger extent in extraocular muscles when compared with facial and tongue muscles. We suggest that the different neurotrophin availability could be related to the particular resistance of extraocular motoneurons to neurodegeneration.

## Introduction

According to the trophic theory of neural connections enunciated by Purves (1990), neurons depend on target-derived trophic support to survive during development. Also, neurotrophins are key actors in the development and maintenance of adult phenotype (Mertz et al., 2000). Thus, these molecules are implicated in regulatory pathways that mediate immature neuron survival (Oppenheim et al., 1992), proper development and migration (Lindholm et al., 1997; Tanaka et al., 2000), as well as the correct establishment of synaptic circuitries (Causing et al., 1997; Morrison and Mason, 1998). Besides, in the adult, neurotrophins have a role in the maintenance of neuron morphological and electrophysiological characteristics, as well as the adequate synaptic functionality, through the regulation of neurotransmitter release and synaptic strength, and the modulation of synaptic input pattern onto neurons (Gonzalez and Collins, 1997; Desai et al., 1999; Novikov et al., 2000; Davis-López de Carrizosa et al., 2009, 2010; Skaper, 2012). The neurotrophin family of trophic factors is composed by four different but structurally related proteins: nerve growth factor (NGF, Levi-Montalcini, 1982), brain derived neurotrophic factor (BDNF; Leibrock et al., 1989), neurotrophin 3 (NT-3; Jones and Reichardt, 1990), and neurotrophin 4 (NT-4; Ip et al., 1992). Neurotrophins are synthesized as pro-proteins, and then, they may be spliced into the mature form, either before or after its exocytosis. In fact, both pro-neurotrophins and their mature forms have physiological effects. They participate in the control of neuron homeostasis by interacting with two different types of receptors. On one hand, neurotrophins may interact with specific Trk receptors (Korsching, 1993; Barbacid, 1994). Thus, dimers of NGF, BDNF, or NT-3 bind TrkA, TrkB, and TrkC, respectively (Klein et al., 1991a,b; Lamballe et al., 1991). NT-4 binds TrkB (Ip et al., 1992), and NT3 may also bind TrkA and TrkB, but with lower affinity. On the other hand, they might interact with the low affinity p75 receptor (Chao and Hempstead, 1995). The interaction of neurotrophin dimers with Trk receptors leads to receptor dimerization, reciprocal phosphorylation of their intracellular tyrosine kinase domain, and the activation of several downstream mechanisms implicated in the regulation of the effects already explained above, including the maintenance of the adult phenotype (Davis-López de Carrizosa et al., 2009).

Extraocular muscles (EOM) are formed by three agonist/antagonist pairs that lead to eye movements around three orthogonal axis of the eye (Büttner and Büttner-Ennever, 2006). In turn, EOM activity depends on motoneurons located in three different brainstem nuclei: (i) the pontine abducens nucleus (whose axons form the VI cranial nerve), (ii) the ponto-mesencephalic trochlear nucleus (IV cranial nerve, and (iii) the mesencephalic oculomotor nucleus (III cranial nerve) (Büttner and Büttner-Ennever, 2006).

The majority of facial muscles are innervated by motoneurons located in the pontine facial nucleus (VII cranial nerve), and tongue muscle are innervated by motoneurons of the hypoglossal nucleus (XII cranial nerve) located at the medulla oblongata.

The role of neurotrophins in the oculomotor system is well documented (for review see Benitez-Temiño et al., 2016). The three high affinity Trk receptors for neurotrophins are expressed by motoneurons in the cat and rat oculomotor system (Benítez-Temiño et al., 2004; Morcuende et al., 2011), suggesting a strong neurotrophin influence on this population of cells. Actually, in rats, the expression of Trk receptors is modulated after lesion (Morcuende et al., 2011), and neurotrophin administration to developing EOM motoneurons reduces lesion-derived cell death (Morcuende et al., 2013). Physiological actions of neurotrophins in the adult oculomotor system have been revealed after exogenous administration to cat axotomized extraocular motoneurons. Strikingly, BDNF and NT-3 exhibit complementary actions on motoneuron firing rate: while BDNF produces the recovery of a firing pattern related to eye position, NT-3 seems to be more implicated in the maintenance of the firing characteristics encoding eye velocity (Davis-López de Carrizosa et al., 2009). NGF, on the other hand, not only avoids the loss of eye-related signals after axotomy, but it also increases firing rates and sensitivities to eye position and velocity (Davis-López de Carrizosa et al., 2010).

Some of these data are surprising since, for instance, TrkA is not expressed by other adult skeletal motoneuron populations (Koliatsos et al., 1991, 1993; Merlio et al., 1992; Henderson et al., 1993; Piehl et al., 1994; Tuszynski et al., 1996). This peculiar relationship with neurotrophins in the adult might be related to the particular resistance of extraocular motoneurons to degeneration during the evolution of some diseases such as ALS. However, it remains to be elucidated whether control extraocular motoneurons are supplied with a higher quantity of neurotrophins when compared with other motor populations. In this project, we have pursued to investigate the actual relevance of neurotrophins by comparing neurotrophin presence in resistant (extraocular) as compared to sensitive (facial and hypoglossal) brainstem motoneuron populations (Nimchinsky et al., 2000). In addition, we have performed Western blot analysis in extraocular, buccinator and tongue muscles, innervated by extraocular, facial, and hypoglossal motoneurons, in an attempt to determine the possible origin of neurotrophins.

## Materials and methods

Experiments were carried out in 13 adult Wistar rats weighing ~250 g, 7 for immunocytochemistry and 6 for Western blot, obtained from our breeding colony (Universidad de Sevilla). All experimental procedures were in accordance with the European Union Directive on the protection of animals used for scientific purposes (2010/63/EU), and Spanish legislation (R.D. 53/2013, BOE 34/11370-421) and the protocol was approved by the ethics committee of the Universidad de Sevilla.

### Immunocytochemistry

Deeply anesthetized animals (sodium pentobarbital, 35 mg/kg, i.p.) were transcardially perfused with 100 ml of 0.9% NaCl followed by 250 ml of 4% paraformaldehyde in 0.1 M sodium phosphate buffer, pH 7.4. Brainstems were dissected, postfixed for 2 h in the same fixative, and cryoprotected by immersion in a solution of 30% sucrose in sodium phosphate buffer. The tissue was then coronally cut at 40 μm thick sections on a cryostat (Leica CM1850, Wetzlar, Germany) and stored in phosphate buffered saline (PBS)-glycerol (1:1). Sections were randomly selected for the immunostaining against each neurotrophin in the different brainstem nuclei.

After several washes in PBS, sections were incubated in a solution of 1% sodium borohydride for 10 min to facilitate antibody penetration. Tissue was then permeabilized in PBS with 0.1% triton (PBS-T) for 30 min, blocked with 10% normal donkey serum (NDS) in PBS-T, for 45 min, and incubated in the corresponding primary antibody solution prepared at 1:400 in PBS-T + 5% NDS + 10% sodium azide for 12 h at room temperature and under gentle agitation (rabbit anti-BDNF, sc-546, rabbit anti-NT-3, sc-547, Santa Cruz Biotechnology, Dallas, TX, USA).

In the case of the immunohistochemistry against NGF, sections were previously incubated in citrate buffer pH 6, 0.01 M, at 40°C for 40 min as an antigen retriever, rinsed in PBS and blocked with 5% NDS in PBS-T at 37°C for 30 min. Tissue was then incubated in a solution containing the primary antibody (rabbit anti-NGF, sc-548, Santa Cruz Biotechnology; 1:250 in PBS-T + 5% NDS + 10% sodium azide), for 48 h at 4°C.

Sections exposed to antibodies directed to either BDNF, NT-3, or NGF were rinsed in PBS-T and incubated in a biotinylated secondary antibody solution (donkey anti-rabbit, Vector, Burlingame, CA; 1:500 in PBS-T) for 2 h, under agitation and room temperature. Tissue was then washed several times in PBS, and immersed for 45 min in a solution containing Streptavidin Cy2 (Jackson ImmunoResearch, West Grove, PA, USA; 1:400 in PBS).

Motoneurons were identified by immunohistochemistry against choline acetyl transferase (ChAT). Briefly, sections were thoroughly washed in PBS-T, blocked with 10% NDS in PBS-T and incubated for 12 h and room temperature in a goat anti-ChAT antibody solution (AB144P, Millipore, Temecula, CA, USA), at 1:500 in PBS-T + 5% NDS + 10% sodium azide. The antibody was then removed, and sections were sequentially washed in PBS-T and incubated in a secondary antibody coupled to TRITC (1:50 in PBS-T, donkey anti-goat, Jackson InmunoResearch) for 2 h. After 3 washes in PBS, slices were mounted on gelatinized glass slides and coverslipped with mounting medium (n-propyl-gallate 0.1 M in PBS-glycerol 1:9).

After immunostaining, a scanning confocal microscope Zeiss LSM 7 DUO (Carl Zeiss, Jena, Germany) was used to visualize the tissue and to obtain and store images. The nuclei of interest were anatomically identified using antibodies directed against ChAT. Off-line image analysis was carried out using ImageJ software (NIH, USA). Using images of Cy2-stained cells, measures of optical density inside the cytoplasm of ChAT-identified motoneurons were taken after BDNF and NT-3 immunostaining. Data were divided by the value of background obtained in the same section. A cell was considered positive when the neurotrophin staining intensity inside the soma was at least five-fold the value of background.

The percentage of positive motoneurons for either BDNF or NT-3 was obtained for each section in each nucleus, and mean values were compared between nuclei (one-way ANOVA, overall level of significance *p* < 0.05). The number of sampled cells depended on the population size. Thus, for trochlear nuclei, the number was between 84 and 117; for abducens nuclei, between 127 and 133; for oculomotor nuclei, between 381 and 468 cells; for hypoglossal nuclei, between 437 and 443, and, finally, for facial nuclei, we sampled 488–520 cells.

We found that NGF immunocytochemistry labeled only the neuropil. Therefore, for measurements of NGF immunostaining intensity we selected squared regions of 30.5 μm side, and optical density was calculated using the ImageJ software. Mean values of NGF optical density were obtained for each nucleus, and compared between the different nuclei (one-way ANOVA, overall level of significance *p* < 0.05). Data correspond to mean ± SEM.

### Western blot analysis

Deeply anesthetized adult rats (sodium pentobarbital, 35 mg/kg, i.p.) were decapitated and extraocular, buccinators, and tongue muscles were dissected. Tissue was homogenized in ice-cold lysis buffer containing a cocktail of proteases and phosphatase inhibitors (10 mM Tris HCl, pH 7.5, 1% NP40, 150 mM NaCl, 1 mM phenylmethanesulfonyl fluoride -PMSF-, 1 μg/ml aprotinin, 10 μg/ml chymostatin, 1 μg/ml leupeptin, 1 μg/ml pepstatin, 5 mM NaF, 20 mM sodium pyrophosphate -Ppi-, 1 mM Na_3_VO_4_). After muscle disruption by sonication, tissue was centrifuged at 13,000 rpm for 30 min. Total protein concentration in the supernatant was determined by the Bradford method (Bradford, 1976), using bovine serum albumin (BSA) as a standard. Proteins were diluted in sample buffer (62.5 mM Tris HCl, pH 6.8, 10% glycerol, 10% SDS, 5% β-mercaptoethanol, 0.05% bromophenol blue), denatured at 95°C for 6 min, and then separated by 15% SDS PAGE (50 μg/lane) and transferred to polyvinylidene difluoride (PVDF) membranes by electroblotting. After blockage of unspecific antigens with 5% BSA for 1 h, blots were incubated overnight at 4°C in a solution with antibodies raised in rabbit against either NGF (1:500), BDNF (1:100), or NT-3 (1:500) diluted in 0.1% TBS-Tween supplied with 5% BSA. Membranes were then incubated in a solution containing the HRP-conjugated anti-rabbit secondary antibody (1:3.000, Vector Labs., Burlingame, CA, USA) prepared in TBS-Tween 0.1% for 90 min at room temperature. The immunoreactions were detected with the Amersham ECL Western blotting kit (GE Healthcare Life Sciences, UK).

Images from the bands were visualized on Image Reader LAS-3000 (Fujifilm), stored and analyzed using Multi-Gauge V.3.0 software. Bands of mature and pro-form of neurotrophins were obtained. For each membrane, data were expressed relative to GAPDH values after background subtraction. Moreover, data were normalized as percentages with respect to the values obtained in the extraocular muscles. Mean values for each neurotrophin and their pro-forms were calculated in each muscle. Comparisons between muscles for the same protein were carried out using one-way-ANOVA test (overall level of significance *p* < 0.05).

## Results

### Neurotrophin presence in oculomotor, trochlear, and abducens nuclei

**Figure 1:**
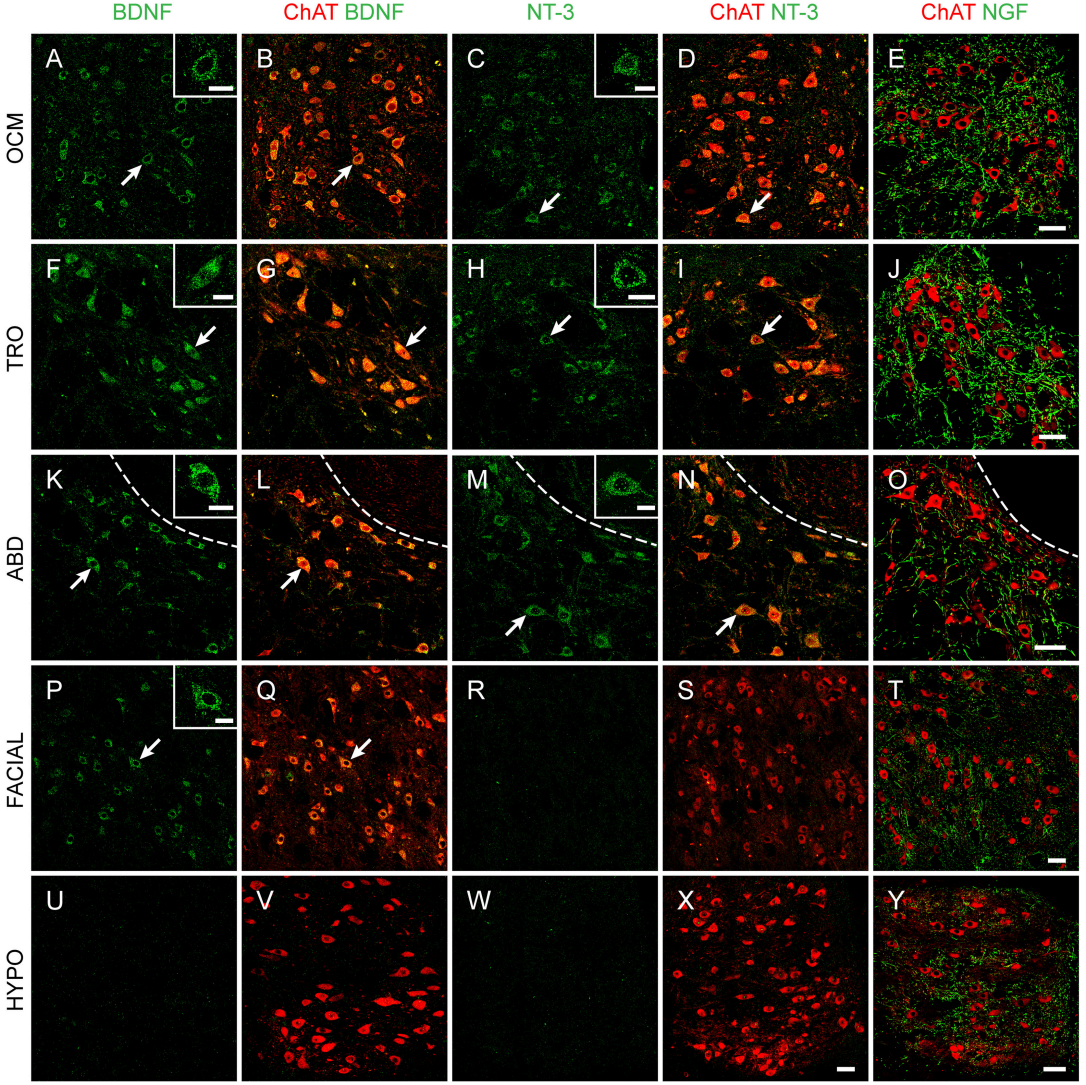
Neurotrophin immunoreactivity in brainstem nuclei. Confocal microscopy images of oculomotor (OCM, **A–E**), trochlear (TRO, **F–J**), abducens (ABD, **K–O**), facial **(P–T)**, and hypoglossal (HYPO, **U–Y**) nuclei illustrating the presence of BDNF (first and second columns), NT-3 (third and fourth columns), and NGF (fifth column) in brainstem motoneurons of adult rats. Motoneurons were identified by ChAT (red). Insets illustrate higher magnification images of neurotrophin-labeled motoneurons in each nucleus. Arrows point to the motoneurons showed in the insets. Dashed lines in **(K–O)** delimit the genu of the facial nerve. Scale bars = 100 μm (in **E** for **A–E**, in **J** for **F–J**, in **O** for **K–O**, in **T** for **P–T**, in **X** for **W,X**, and in **Y** for **U,V**, and **Y**). Inset scale bars = 25 μm.

Immunohistochemistry against ChAT allowed the unambiguous identification of extraocular motoneurons, located in the brainstem (oculomotor, trochlear, and abducens nuclei, shown in Figure 1, first three rows). This method also allowed distinguishing motoneurons from internuclear neurons, which do not express ChAT.

**Figure 2:**
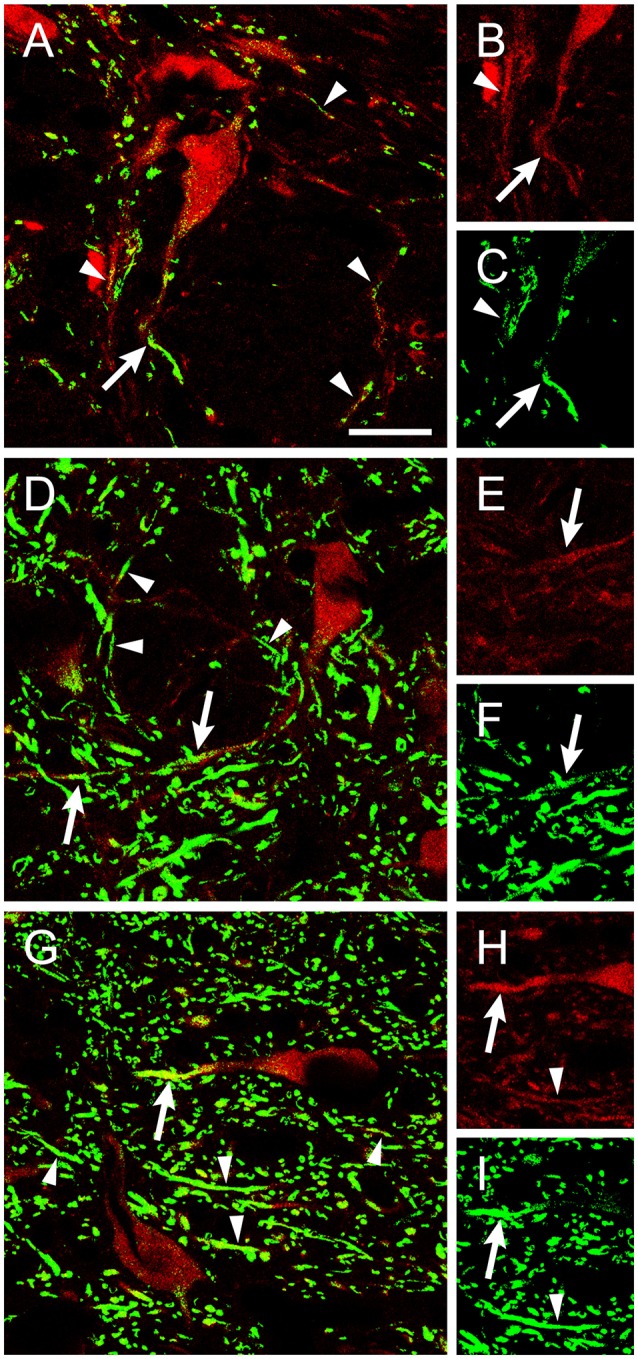
ChAT-positive dendrites were stained using an antibody against NGF. Confocal images of abducens **(A–C)**, facial **(D–F)**, and hypoglossal **(G–I)** nuclei showing ChAT-positive dendrites (red) that colocalized with NGF (green). Arrows point to NGF-positive dendrites connected to the soma of ChAT-identified motoneurons (red). Arrowheads point to dendrite-like ChAT-positive processes stained against NGF. Right column shows separately the ChAT and NGF staining for the same dendrite. Scale bar = 25 μm.

The vast majority of extraocular motoneurons, located in any of the three oculomotor-related nuclei, were positive for BDNF and NT-3, as revealed by the presence of a diffuse staining in the cytoplasm of extraocular motoneurons (Figures 1A,F,K for BDNF, Figures 1C,H,M for NT-3). Neither internal compartments in the cytoplasm nor plasmatic membrane could be distinguished, although the nucleus remained completely unstained (Figure 1, arrows). However, neurotrophin-negative ChAT-identified motoneurons could be scarcely observed. In contrast to BDNF and NT-3, motoneuron somata were not stained against NGF. Instead, NGF-positive dendrites, demonstrated in many instances as originating from motoneuron cell bodies (Figures 2A–C), were distributed throughout the neuropil (Figures 1E,J,O). The three extraocular motor nuclei showed the same pattern of staining.

### Neurotrophin presence in facial and hypoglossal nuclei

We also aimed to describe neurotrophin presence in brainstem motor nuclei that result damaged during the degenerative processes occurring in ALS. A high proportion of facial motoneurons were positive for BDNF (Figures 1P,Q), with scarce ChAT-positive BDNF-negative neurons distributed scattered in the nucleus. On the contrary, only scant facial motoneurons were positive for NT-3 (Figures 1R,S). No facial motoneuron somata were positive for NGF, although, as was the case in the oculomotor system, NGF-positive processes were found throughout the neuropil (Figures 1T,2D–F).

Regarding hypoglossal motoneurons, rare cells were positive for BDNF (Figures 1U,V) or NT-3 (Figures 1W,X). Again, NGF-positive processes were detected in the neuropil (Figures 1Y,2G–I). As described for extraocular motoneurons, BDNF and NT-3, when present in facial and hypoglossal motoneurons, were detected homogenously distributed in the cytoplasm.

### Quantitative analysis of neurotrophin staining reveals differences between oculomotor and non-oculomotor neurons

**Figure 3:**
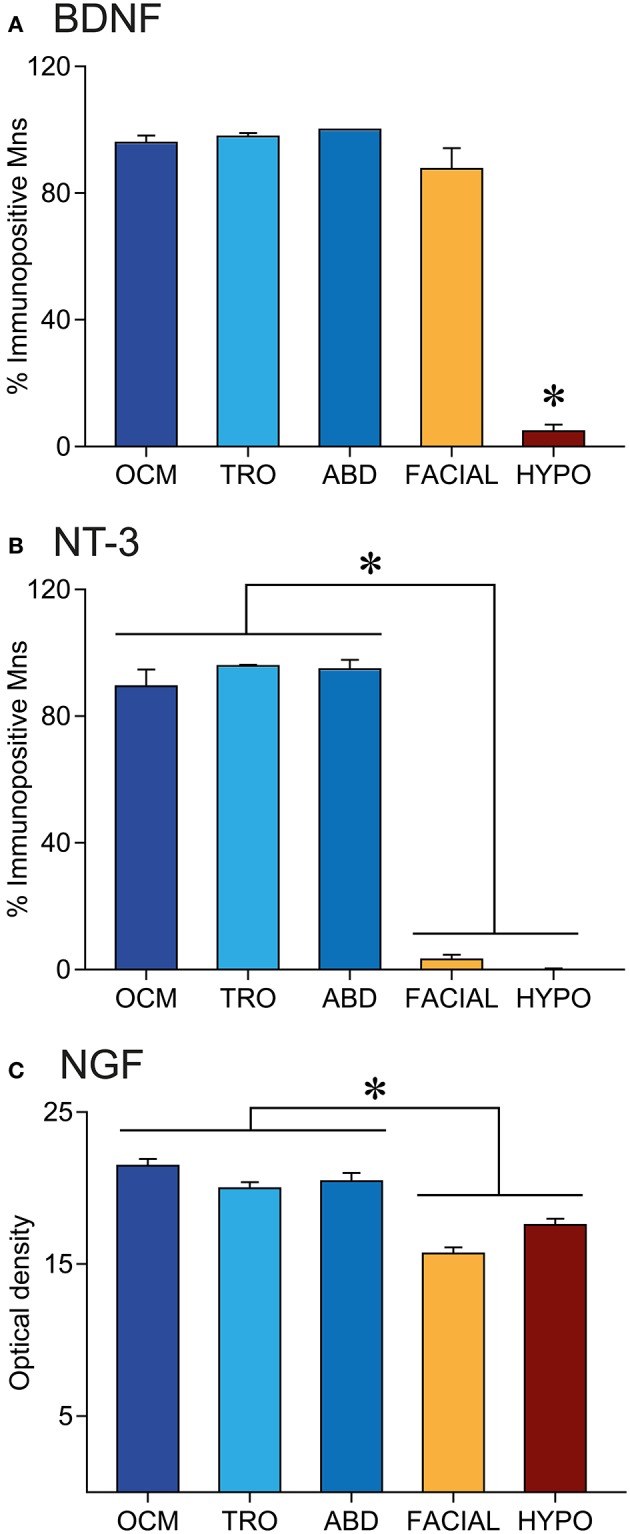
Neurotrophin immunoreactivity is higher in extraocular than in non-extraocular brainstem motoneurons. Quantification of the percentage of BDNF **(A)** or NT-3 **(B)** immunopositive motoneurons and NGF staining in the neuropil **(C)** of brainstem motor nuclei. Bars represent mean ± SEM. The asterisks represent significant differences between groups (one-way ANOVA test followed by Holm–Sidak method for multiple pairwise comparisons, *p* < 0.001).

The percentage of positive cells with respect to the total number of motoneurons within an extraoculomotor nucleus was similar when compared between BDNF and NT-3. In the case of the oculomotor nucleus, 95.9 ± 2.2% of motoneurons were BDNF-positive (*n* = 381), while 89.4 ± 5.3% (*n* = 468) were positive for NT-3. The percentage of positive motoneurons for BDNF and NT-3 was 97.8 ± 1.1% (*n* = 84) and 95.8 ± 0.4% (*n* = 117), respectively, in the trochlear nucleus, and 100% (*n* = 127) and 94.8 ± 2.95% (*n* = 133) in the abducens nucleus (Figures 3A,B, blue bars). No significant differences were detected between the neurotrophins BDNF or NT-3 either within the same nucleus (*t*-test), or between the three extraoculomotor nuclei for the same neurotrophin (one-way ANOVA test).

On the other hand, the quantitative analysis showed differences between extraocular motoneurons, located in any of the three extraoculomotor nuclei, and other brainstem motor system motoneurons (Figure 3). As stated above, the percentage of BDNF-positive and NT-3 positive motoneurons was similar in the three extraocular nuclei (Figures 3A,B, blue bars). However, although the percentage of BDNF-positive facial motoneurons was also similar to that of extraocular motoneurons (87.6 ± 6.5%, *n* = 520), only 4.8 ± 2.1% (*n* = 443) of hypoglossal motoneurons were positive for BDNF, which was significantly different when compared with any of the other 4 nuclei (one-way ANOVA test followed by Holm-Sidak method for multiple pairwise comparisons, *p* < 0.001, Figure 3A). Regarding NT-3, positive-motoneurons represented only a 3.2 ± 1.4% (*n* = 488) and a 0.2 ± 0.2% (*n* = 437) of facial and hypoglossal motoneurons, respectively, which was significantly different from values obtained in the extraocular nuclei (one-way ANOVA test, followed by Holm–Sidak method for multiple pairwise comparisons, *p* < 0.001, Figure 3B).

We also measured optical density in the neuropil of brainstem sections immunostained against NGF. The optical density values in the oculomotor, trochlear, and abducens nuclei were, respectively, 21.4 ± 0.4, 20.0 ± 0.4, and 20.4 ± 0.5, which were statistically similar between them (Figure 3C, blue columns). Values obtained in the facial and hypoglossal nuclei (15.7 ± 0.4 and 17.6 ± 0.4, respectively) were significantly lower when compared with those obtained in the oculomotor system (one-way ANOVA test, followed by Holm–Sidak method for multiple pairwise comparisons, *p* < 0.001, Figure 3C).

### Presence of neurotrophins in cranial muscles

**Figure 4:**
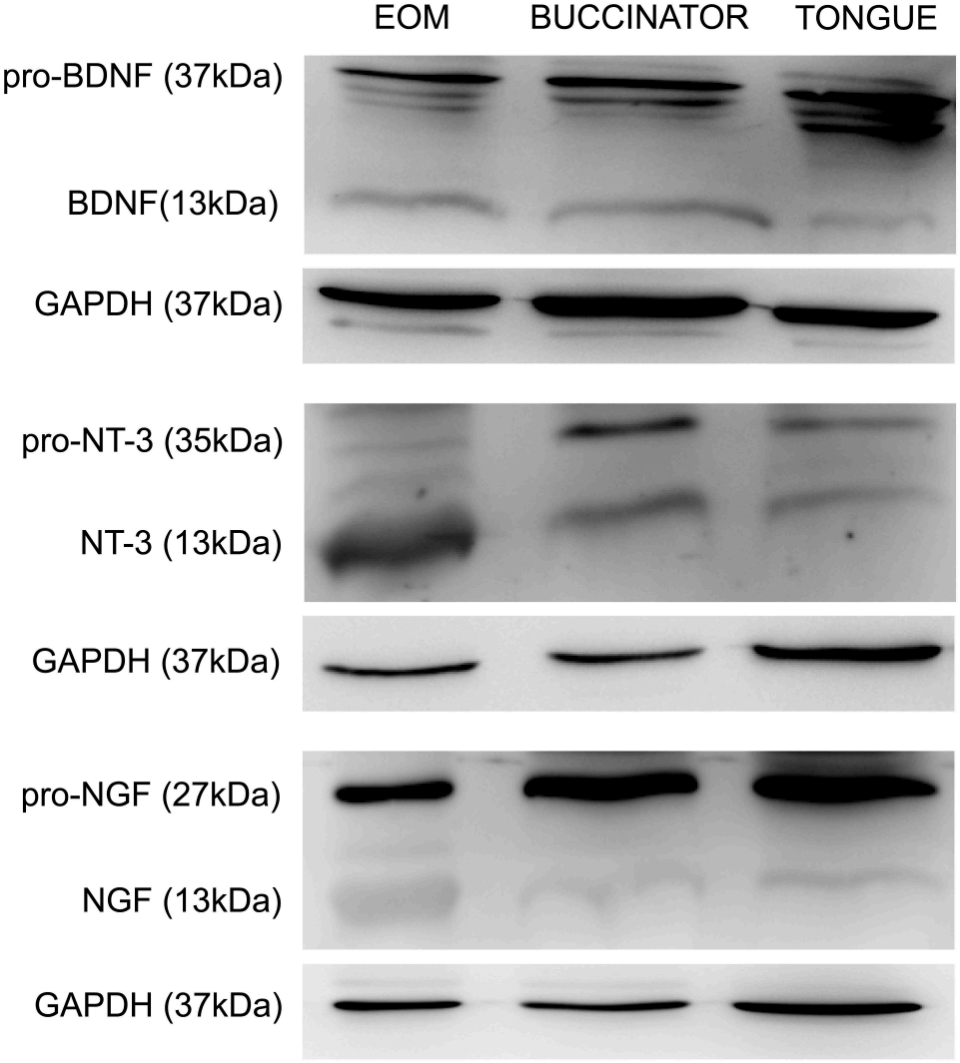
Representative Western blots of tongue, buccinator and extraocular muscles. Immunoblotting using antibodies against BDNF, NT-3, and NGF in the muscles showed a positive labeling for both the pro-neurotrophin (37, 35, and 27 kDa for BDNF, NT-3, and NGF, respectively) and the mature forms (13 kDa). Other molecular weight isoforms also appeared stained, probably representing immature forms of these trophic factors. GAPDH immunoblotting was used as a loading control. EOM, extraocular muscles.

We studied the presence of neurotrophic factors, BDNF, NT-3, and NGF, in the extraocular, buccinators and tongue muscles of adult rats by means of protein immunoblot analysis. The six extraocular muscles were processed in the same sample. We found that the three neurotrophins were present in all muscles analyzed. Bands of 13 kDa were obtained corresponding to the mature forms of BDNF, NT-3, and NGF. Higher molecular weight isoforms were also detected, corresponding to precursor peptides. We focused in the pro-neurotrophin band, with a molecular weight of 37, 35, and 27 kDa for BDNF, NT-3, and NGF, respectively (Figure 4).

**Figure 5:**
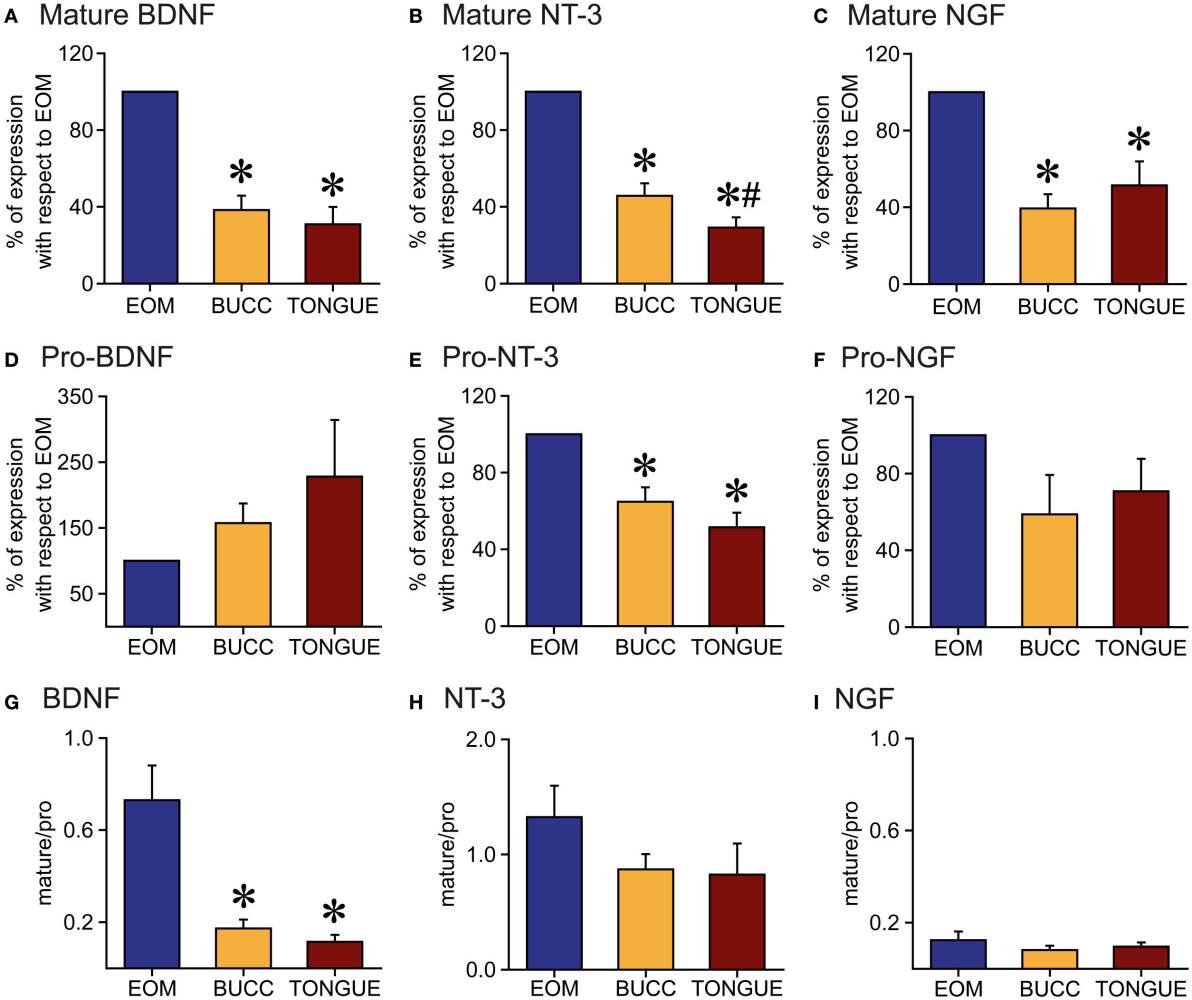
Quantification of neurotrophin protein in the muscles. **(A–C)**: Mature BDNF **(A)**, NT-3 **(B)**, and NGF **(C)** forms presented higher densitometric values in extraocular muscles (EOM) than in buccinators (BUCC) or tongue muscles. **(D–F)**: Immunoblot analysis of immature BDNF **(D)** and NGF **(F)** isoforms revealed no differences between the three muscles. The amount of Pro-NT-3 **(E)** in extraocular muscles was larger than that observed in non-extraocular muscles. **(G–I)**: Mature/pro-form ratio for BDNF **(G)**, NT-3 **(H)**, and NGF **(I)**. Note the ratio was lower than 1 for all neurotrophins in all muscles except for NT-3 in the EOM. Asterisks represent significant differences with extraocular muscles, whereas the hashtag indicates differences with buccinator (one-way ANOVA test followed by Holm–Sidak method for multiple pairwise comparisons, *p* < 0.001).

The total amount of BDNF, NT-3, and NGF protein was quantified by densitometric analysis and values were normalized to EOM data. Thus, EOM, compared to buccinator and tongue muscles, presented a higher amount of mature BDNF (100, 38.3 ± 7.5, 30.9 ± 9.0, Figure 5A), NT-3 (100, 45.7 ± 6.5, 29.1 ± 5.3, Figure 5B), and NGF protein (100, 39.4 ± 7.4, 51.4 ± 12.5, Figure 5C), respectively. The amount of pro-neurotrophin in extraocular and non-extraocular muscles varied depending on the neurotrophin tested. Thus, whereas no differences were detected between the total amount of pro-BDNF in the three samples (100, 157.3 ± 29.7, 228.0 ± 86.0, Figure 5D), NT-3 (100, 64.8 ± 7.5, 51.5 ± 7.6, Figure 5E), and NGF (100, 58.7 ± 20.5, 70.8 ± 16.9, Figure 5F) immature forms were significantly more abundant in extraocular than in non-extraocular muscles (one-way ANOVA test followed by Holm–Sidak method for multiple pairwise comparisons, *p* < 0.001).

The statistical analysis of the mean ratio of the mature and pro-form peptides of each neurotrophin demonstrated an uppermost quantity of immature BDNF in the three muscles studied, but we observed a significant smaller ratio mature/immature in the non-extraocular muscles than in EOM (0.7 ± 0.1, 0.2 ± 0.04, 0.1 ± 0.03, one-way ANOVA test followed by Holm–Sidak method for multiple pairwise comparisons, *p* < 0.001, Figure 5G). For NT-3, the ratio mature/pro-form was similar between the different muscles (1.3 ± 0.3, 0.9 ± 0.1, 0.8 ± 0.3, Figure 5H). Regarding NGF, we did not find differences in the ratio mature/immature forms between extraocular, buccinators, and tongue muscles (0.1 ± 0.04, 0.1 ± 0.02, 0.1 ± 0.02, Figure 5I). For NT-3 protein, we measured a higher quantity of mature NT-3 isoform in the EOM, which was not found in buccinator and tongue muscles (Figure 5H). In the case of NGF protein (Figure 5I), the amount of immature form was higher than the immature form in all muscles.

## Discussion

**Figure 6:**
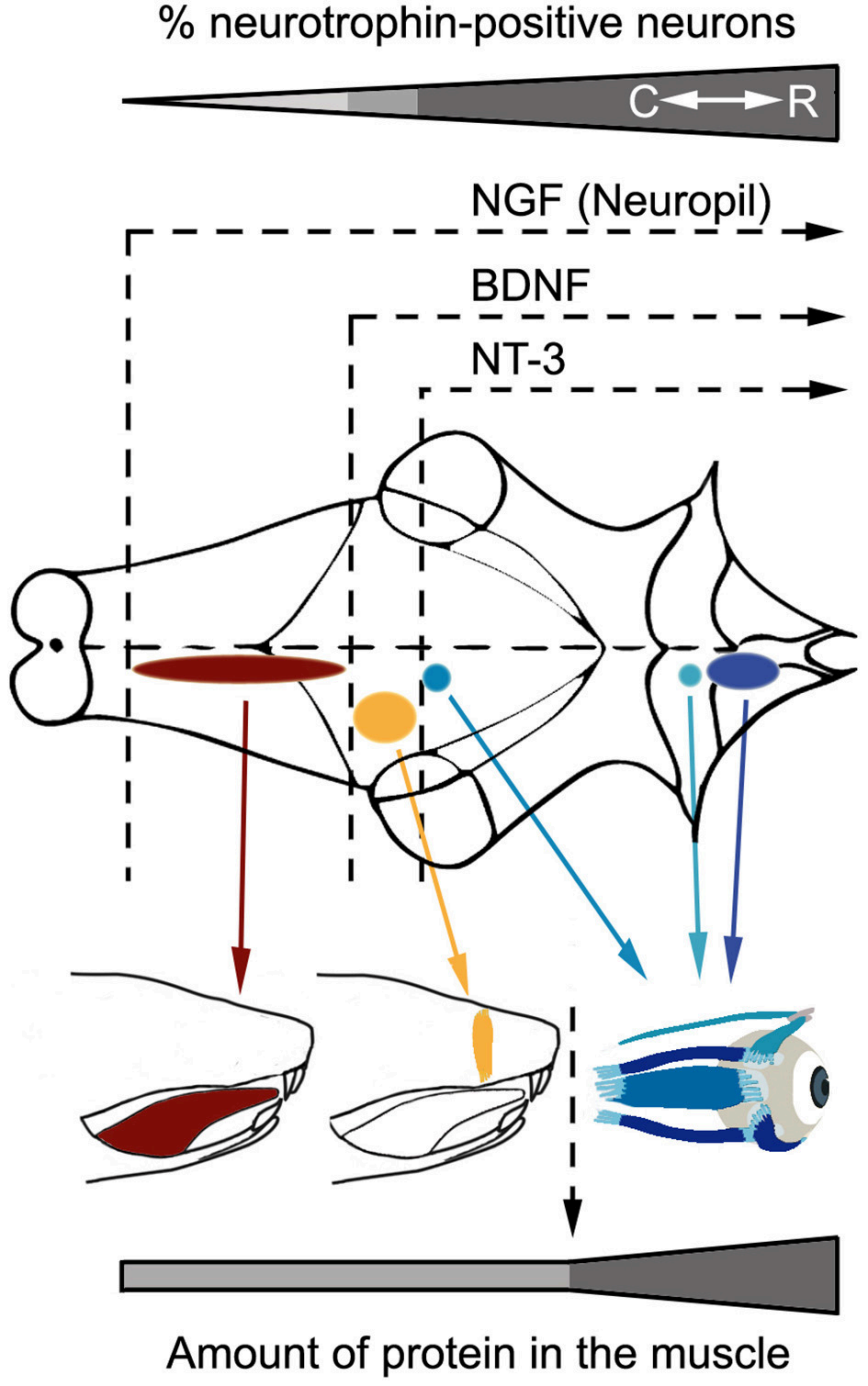
Rostro-caudal gradient of neurotrophin influence in the analyzed brainstem motor systems. The scheme shows neurotrophin immunoreactivity pattern in motoneurons and muscles belonging to the oculomotor system (blue), facial motor system (yellow), and hypoglossal motor system (red). Note that neurotrophins are less abundant as motor systems are located more caudally in the brainstem, both in terms of neurotrophin diversity (motoneurons) or quantity (muscles). C, Caudal; R, Rostral.

The main objective of the present work was to reveal the expression pattern of neurotrophins in the oculomotor system of the adult rat, as well as to compare these results with those obtained from other brainstem motor systems, such as facial and hypoglossal. The present results show a rostro-caudal gradient in neurotrophin expression in both innervating and target cells (Figure 6). These results are in accordance with data obtained from adult human (Tang et al., 2010) and rat brains (Li et al., 2001) and muscles (Nosrat et al., 2000; Harandi et al., 2014).

The presence of the three neurotrophins in the adult control oculomotor system is in good agreement with previous results showing that extraocular motoneurons express the three high affinity Trk receptors for neurotrophins (Benítez-Temiño et al., 2004; Morcuende et al., 2013). In fact, the exogenous administration of BDNF or NT-3 exerts complementary physiological actions on axotomized adult cat abducens motoneurons (Davis-López de Carrizosa et al., 2009), and NGF restores and even increments the firing pattern related to eye movements (Davis-López de Carrizosa et al., 2010). Moreover, the present results show that, indeed, neurotrophins are present constitutively in the adult oculomotor system. Possible implications of neurotrophin expression in the oculomotor system, and the relevance of the differences observed with respect to other brainstem motor nuclei, are discussed in the following sections.

### Presence of BDNF in extraocular and facial but not hypoglossal motoneurons

BDNF was present in almost all rat extraocular motoneurons, located either in the oculomotor, trochlear or abducens nuclei. Accordingly, BDNF was also present in the extraocular muscles.

The presence of BDNF in motoneuron somata is not a direct indicator of the expression of the BDNF gene, since it is well-known that neurotrophins may act via retrograde, anterograde, paracrine, besides autocrine, pathways in motor neurons (DiStefano et al., 1992; Korsching, 1993; Reynolds et al., 2000). In the case of extraocular and facial motoneurons, it must be considered that, first, BDNF protein and mRNA is present in extraocular and buccinator muscles (Koliatsos et al., 1993; Gómez-Pinilla et al., 2001) and in the neuromuscular junction of extraocular muscles (Harandi et al., 2016), and second, extraocular and facial motoneurons express the TrkB receptor (Merlio et al., 1992; Yan et al., 1994; Morcuende et al., 2013). Moreover, both populations are sensitive to retrogradely administered BDNF after lesion (Yan et al., 1994; Davis-López de Carrizosa et al., 2009). Consequently, BDNF protein present in the motoneurons could have been synthesized by the target muscle and retrogradely transported toward de soma.

However, it cannot be discarded that the BDNF present in extraocular motoneurons could have other additional sources. Thus, direct neurotrophin interaction with receptors located in the soma or dendrites of both developing and mature neurons have already been described, demonstrating anterograde (Von Bartheld et al., 1996; Fawcett et al., 1998), paracrine (Robinson et al., 1996), and even autocrine (Miranda et al., 1993; Acheson et al., 1995; Davies, 1996; Buck et al., 2000) actions of BDNF.

Regarding facial motoneurons, although the percentage of immunopositive-cells was similar to that obtained in the extraocular motor system, our results show that the amount of BDNF protein in the facial muscle was lower than that observed in extraocular muscles, and the ratio mature/pro-BDNF was lower, indicating a higher quantity of the immature form of BDNF in the buccinator muscle, which has a lesser ability to activate the TrkB pathway than the mature form (Mowla et al., 2001). Therefore, other possible sources of BDNF for facial motoneurons should also be considered. In fact, it has been postulated that neurotrophins arriving to different compartments of the cell could participate in different cell functions (Heerssen and Segal, 2002), so different sources of neurotrophins would not be redundant.

Regarding the anterograde pathway, two major afferences to extraoculomotor nuclei arise from pontine and medullary vestibular and prepositus hipoglossi neurons (Büttner and Büttner-Ennever, 2006). The presence of BDNF protein and the expression of the mRNA have been demonstrated in adult rat vestibular and prepositus neurons (Li et al., 2001). Since vestibular cells also project toward facial motoneurons (Shaw and Baker, 1983), they could also serve as an anterograde source of neurotrophins for these cells.

Besides, BDNF could have a paracrine origin. In this case, glial cells are the main candidates, since BDNF is released by astrocytes under control conditions (Takemoto et al., 2015). However, although in the present study a clear identification of glial cells was not performed, we did not observe any BDNF positive-cell that could resemble the shape and size of astrocytes or microglia, suggesting that glia is probably not an important source of BDNF in oculomotor or facial motor systems. Finally, the origin of BDNF could be autocrine, as suggested for spinal motoneurons (Nishio et al., 1998). However, further research is needed to test whether neurotrophin mRNA synthesis occurs in motoneurons located at the facial and extraocular motor nuclei.

Taken together, present and previous data suggest that even considering other alternative sources of neurotrophins, retrograde BDNF is needed for extraocular motoneurons to maintain their normal adult properties (Davis-López de Carrizosa et al., 2009). In contrast, the anterograde/paracrine/autocrine routes seem to be necessary to explain the presence of BDNF in facial motoneurons.

On the other side, the vast majority of hypoglossal motoneurons were BDNF immunonegative. These cells only express the NT-3 receptor, TrkC, but neither TrkA nor TrkB (Merlio et al., 1992), and thus are likely insensitive to BDNF. In fact, BDNF protein present in the muscle could have not been originated in tongue muscle fibers, since no BDNF mRNA has been found in these muscle fibers, whereas sensitive taste buds do express this neurotrophin (Nosrat et al., 2000), so its function in the tongue could be independent of the tongue motor system. Although, special care was taken to avoid taste buds inclusion in the samples, the possibility of some degree of contamination cannot be discarded. Another possibility is that tongue BDNF has an endothelial origin, as vascular endothelial cells express this trophic factor (Nakahashi et al., 2000). If this was the case, then BDNF protein present in tongue muscle fibers could have a paracrine origin, and its function would be restricted to the muscle, and not to hypoglossal motoneurons. However, and in opposition to this hypothesis, retrograde transport of exogenous BDNF can restore ChAT expression in axotomized hypoglossal motoneurons (Wang et al., 1997), indicating that, at least after axotomy, this neurotrophin is trophic for hypoglossal motoneurons.

### NT-3 is present in the extraocular motor system

As was the case for BDNF, NT-3 was present in most of extraocular motoneurons, as well as in EOM. These data, together with the facts that, first, extraocular motoneurons express TrkC (Morcuende et al., 2011), and second, retrogradely-driven NT-3 is necessary to maintain certain motoneuron properties (Davis-López de Carrizosa et al., 2009), indicate a retrograde action of NT-3 in the extraocular motor system. These results are in accordance with those obtained by Harandi et al. (2014, 2016), in which they described NT-3 expression in EOM and NT-3 presence at the neuromuscular junction of EOM obtained from control mice.

Only scarce facial motoneurons, and no hypoglossal motoneuron, were immunoreactive to NT-3, even though both populations express the high affinity receptor for NT-3 (Merlio et al., 1992), and NT-3 was present in both target muscles (buccinator and tongue muscles, respectively), although to a lower extent when compared with EOM. The most likely explanation of this apparent contradiction between the extremely low presence of NT-3 in facial and hypoglossal motoneurons, compared with their respective muscles, is that muscle-derived NT-3 could bind TrkC receptors located in the axon terminal and exert its function locally at the axonal level, without being transported to the soma, or that muscle-derived NT-3 might signal the cell body through intermediary effector molecules. However, in agreement with these results, a scarce or null effect of exogenous NT-3 in regulating ChAT expression has been shown after facial or hypoglossal nerve section (Tuszynski et al., 1996; Fernandes et al., 1998). These results would imply a low responsiveness of these motoneurons to NT-3.

Thus, the fact that, first, NT-3 is more abundant in EOM muscles than in tongue or buccinator muscles, and second, this neurotrophin in necessary for the maintenance of extraocular motoneuron adult characteristics (Davis-López de Carrizosa et al., 2009), could point to a higher relevance of retrogradely-driven NT-3 in the maintenance of oculomotor function, when compared with the other two motor systems.

### NGF is present in the neuropil of brainstem motor nuclei

The antibody against NGF produced a pattern of labeling that differed radically from that obtained against the other two neurotrophins. In the five nuclei analyzed, NGF was absent from motoneuron somata. Instead, long NGF-positive, dendrite-like processes could be observed dispersed throughout the nucleus neuropil and in several instances could be demonstrated arising from motoneurons.

Facial and hypoglossal motoneurons, like spinal motoneurons, do not express the TrkA receptor (Koliatsos et al., 1991, 1993; Merlio et al., 1992; Henderson et al., 1993; Piehl et al., 1994; Tuszynski et al., 1996). Considering the low levels of mature NGF found in the buccinator and tongue muscles, together with the lack of TrkA expression by their innervating motoneurons, the absence of NGF in facial and hypoglossal motoneurons somata is not surprising. On the contrary, experiments using immunohistochemistry and *in situ* hybridization in extraocular motoneurons have shown not only TrkA expression under control conditions (Benítez-Temiño et al., 2004; Morcuende et al., 2011), but also a modulation of its expression in response to axotomy (Morcuende et al., 2011). The amount of mature NGF in EOM was higher than that observed in buccinator and tongue muscles. Thus, we would expect to find retrogradely-transported NGF in extraocular motoneuron soma. In accordance with this hypothesis, extraocular motoneurons have been proven to be sensitive to NGF after axotomy and exogenous administration of this neurotrophin through their proximal axonal stumps (Davis-López de Carrizosa et al., 2010).

Regarding the possible origin of the NGF observed in the neuropil, this neurotrophin could arise from different unknown sources. On one side, NGF positive-processes could belong to astrocytic or microglial cells, that express constitutively this neurotrophin (Sofroniew et al., 2001). However, the shape and the size of NGF-positive processes were not indicative of glial origin. On the other, NGF could follow an anterograde route, being expressed at extraocular, facial and hypoglossal afferences. Finally, NGF could be synthesized by motoneurons and released from their dendrites acting trophically to afferences. Indeed, our data suggest this possibility due to the colocalization observed in motoneuron dendrites between ChAT and NGF. In comparison with facial and hypoglossal nuclei, the present results show a stronger staining after NGF immunocytochemistry in the extraoculomotor nuclei. These data, together with the constitutive expression of TrkA in extraocular motoneurons–a receptor that is not present in other motoneuronal types-, point to a higher potentiality of NGF as a physiological and metabolic mediator of oculomotor function when compared with facial and hypoglossal motor systems.

### Possible influence of neurotrophins in the resistance to ALS of extraocular motoneurons

ALS affects mainly motoneurons, with no discrimination between cranial or spinal positions. However, brainstem motoneurons innervating extraocular muscles, and sacral motoneurons projecting on pelvic floor muscles, exhibit a much stronger resistance to degeneration (Ohki et al., 1994; Nimchinsky et al., 2000). This particular resistance might be due to distinct, special features affecting motoneurons, their target muscles, or both. Unrevealing these possible differences could represent an important step toward the understanding of ALS neurodegenerative mechanisms, opening new lines of research to improve motoneuron survival.

Interestingly, the oculomotor system is also different from other motor systems in its sensitivity to neurotrophins. Thus, only extraocular motoneurons express the TrkA receptor for NGF, which is absent from other brainstem and spinal motoneurons (Koliatsos et al., 1991, 1993; Merlio et al., 1992; Henderson et al., 1993; Piehl et al., 1994; Tuszynski et al., 1996). Extraocular motoneurons also respond to neurotrophins after lesion by recovering their normal discharge activity and synaptic inputs (Davis-López de Carrizosa et al., 2009, 2010). Due to the well-known ability of neurotrophins to increase motoneuron survival during development and after lesion, trophic factors are good candidates to be implicated in motoneuron survival/degeneration during the progression of ALS.

In fact, *post-mortem* analysis of spinal cords obtained from ALS patients have demonstrated a decreased amount of NT-3 and BDNF in spinal motoneurons (Duberley et al., 1997; Nishio et al., 1998), as well as an increase in the quantity of non-phosphorylated TrkB (Mutoh et al., 2000), pointing to a diminution in trophic signaling in degenerating motoneurons during ALS progression in humans. It would be interesting to elucidate whether extraocular motoneurons alter neurotrophin expression during ALS progression, or instead maintain their BDNF and NT-3 signaling.

The effect of exogenous neurotrophin administration in ALS murine models also highlights the potential therapeutic use of these molecules, although paradoxical results obtained by different groups invite to be critical. Thus, while some groups have reported neither effects of BDNF and NT-3 on motoneuron survival when exposed to glutamate toxicity in culture (Corse et al., 1999), nor an increase in lifespan in ALS mouse models (Park et al., 2009), other groups have demonstrated an improved motor function and an increased survival of motoneurons treated with BDNF or NT-3 in different murine models of ALS (Mitsumoto et al., 1994; Ikeda et al., 1995; Haase et al., 1997; Kaal et al., 1997). However, neurotrophin therapy in ALS patients has not revealed to be useful in the treatment of this disease (Bradley, 1999; Ochs et al., 2000; Henriques et al., 2010).

Altogether, due to the numerous trophic effects described for neurotrophins in motoneurons, and considering that, first, extraocular motoneurons and muscles are enriched in these molecules in contrast to other cranial motor systems, and, second, that extraocular motoneurons are less vulnerable to ALS, we suggest that the high presence of neurotrophins in the extraoculomotor system might be linked to their higher resistance to this disease.

## Author contributions

All authors contributed to the design of the experiments and the interpretation of the results. BB wrote the manuscript. RH and BB carried out immunocytochemistry experiments, analyzed the results, and prepared the figures. SS and SM set up the western blot techniques, performed the experiments and analyzed the results. AP and Rd critically revised the manuscript and participated in the design and correction of the figures.

### Conflict of interest statement

The authors declare that the research was conducted in the absence of any commercial or financial relationships that could be construed as a potential conflict of interest.
